# Contraceptive use and associated factors among sexually active reproductive age HIV positive women attending ART clinic at Felege Hiwot Referral Hospital, Northwest Ethiopia: A cross-sectional study

**DOI:** 10.1016/j.heliyon.2020.e05653

**Published:** 2020-12-14

**Authors:** Tilahun Tewabe, Tilksew Ayalew, Abdulhakim Abdanur, Demoze Jenbere, Mastewal Ayehu, Girma Talema, Eden Asmare

**Affiliations:** a Department of Pediatrics and Child Health Nursing, College of Medicine and Health Science, Bahir Dar University, Bahir Dar, Ethiopia; b Department of Maternal and Reproductive Health Nursing, College of Medicine and Health Science, Bahir Dar University, Bahir Dar, Ethiopia; c Bahir Dar University College of Medicine and Health Sciences, Bahir Dar, Ethiopia; d Department of Midwifery, College of Medicine and Health Science, Bahir Dar University, Bahir Dar, Ethiopia

**Keywords:** Public health, Infectious disease, Immunology, Women's health, Reproductive system, Clinical research, Contraceptive use, Human immune virus positive women, Bahir dar, Ethiopia, Reproductive health

## Abstract

**Background:**

Contraception helps prevent unplanned pregnancies and mother to child Human Immune Virus (HIV) transmission among human immune virus positive women. Ethiopia has made remarkable progress in increasing contraceptive use rate but there is still a disparity of contraceptive use within the country. Although there were some studies about contraceptive use in Ethiopia, evidences about contraceptive use among sexually active HIV positive women was limited. Understanding the extent of and barriers in Ethiopia is important for learning how to best improve level of contraceptive use. Therefore, this study aimed to assess contraceptives use and associated factors among HIV positive sexually active women at anti-retroviral therapy clinic at Felege Hiwot Referral Hospital ART clinic in Bahir Dar, Ethiopia.

**Method:**

A facility-based cross-sectional study was conducted from June 01–30, 2018 among (n = 308) randomly selected HIV positive women at Felege Hiwot Referral Hospital. Data were collected using a pretested interviewer-administered questionnaire and analyzed using SPSS version 20. Survey logistic regression analysis was employed to identify determinants of contraceptive use. Statistical significance was declared at p-value <0.05.

**Results:**

Out of three hundred eight participants, 118 (38.3%) reported contraceptive use at the time of the study. Injectable is the most preferred (43.5%) contraceptive method. Participants with age 15–34 years (AOR = 3.09, 95%CI: 1.59–5.99), disclosed their status to sex partner, (AOR = 2.7, 95%CI: 1.14–6.66), had history of contraception use; (AOR = 3.36, 95%CI: 1.68–6.74), were sexually active (AOR = 5.45, 95%CI: 2.72–10.91) had higher odds of contraceptive use. However, participants who had drinking habit (AOR = 4.35, 95%CI: 1.82–10.38) had lower odds of contraceptive use.

**Conclusion:**

A significant proportion of HIV positive women had low level of contraceptive use that was lower than the national recommended level. Participants with younger age (15–34years), who disclosed HIV status to sex partner, had history of contraceptive use, and who were sexually active six months prior to the study were more likely to use contraception. However, participants who had drinking habit were less likely to use contraception. These results suggest that multi-sectorial and multi-disciplinary approaches are needed to increase contraceptive use in the HIV positive women.

## 1 Introduction

Even though the global community is working to end HIV/AIDS epidemic by 2030, it remains a major global epidemic in post millennium development era [1,2]. It remains the leading cause of illness and death throughout the world. Since the start of the epidemic, around 76.1 million peoples infected and 35 million people died of AIDS related illness [3]. In 2017, 36.9 million people were living with HIV; 1.8 million people were newly infected worldwide. From these, 18.2 million were women of reproductive age and 1.8 were children under-15 years old [2]. In Ethiopia, there were an estimated total 722,248 people infected with HIV, 14,872 annual AIDS related deaths, 22,827 new HIV infection in 2017 [4]. In Sub-Saharan Africa, women, infants and young children account for more than 60 percent of new HIV infections [5]. According to a 2013 family health international report, there were about 100,000 pregnancy of HIV positive women and 12,000 HIV positive births annually, mostly unintended pregnancies in Ethiopia [6].

Family planning is one of a key intervention to curve HIV/AIDS epidemic [7,8]. It allows people including HIV positive women to make informed choices about their sexual and reproductive health through information, education and contraceptive methods [8-11]. HIV positive women, like other women, may wish to plan pregnancy, limit their family and avoid pregnancy [12]. In the time when approximately 36.9 people are living with HIV/AIDS [2] and women of reproductive age accounts for nearly half of the infected population, family planning plays a critical role in averting HIV/AIDS epidemic by reducing unintended pregnancy [9,11]. Despite, unmet need of for family planning remains high in most of the world countries especially in low and middle income countries [13]. By the year 2015–2019, the proportion of unintended pregnancy ending in abortion was 61% increased by 10% in 2000–2004 which was 51% [14]. In 2012, there were approximately 85 million unintended pregnancies [9]. Besides, 190 million reproductive age women worldwide who wanted to avoid pregnancy were not using any method of contraception in 2019 [13].

Unintended pregnancy is a major root cause for abortion. Each year, about 22 million unsafe abortions occur contributing for 67,000 annual maternal deaths worldwide [10,11]. In Africa, nearly 5.5 million women have unsafe abortion. Nearly, 36,000 of these women die of the abortion procedure [14,15]. The situation is worse in Ethiopia. Low levels of contraceptive use including in HIV positive women lead to high level of unintended pregnancy [15].

Unsafe abortion is a major cause to the sustained high global maternal mortality and morbidity. Legal abortion and access to reproductive health and contraceptive have been pointed out as a vital to reduce abortion rate [16]. Accordingly, Ethiopia has liberalized its abortion law in 2005, which had previously allowed the procedure only to save the life of a woman or protect her physical health. Now abortion is legal in Ethiopia in case of rape, incest, fetal impairment and if the fetus has severe defect, or if a girl is under the age of 18 years old [15,17]. Despite, a substantial number of abortions continue to occur outside health facilities unsafely [18].

Family planning has a tremendous benefit to the world population. Advancing human rights, reducing poverty, improving nutrition, saving millions of lives, advancing gender equality and empowerment contributing to the economic growth are some of the benefits of family planning [9,19].

Many factors affect contraceptive use. Economic factors, Cultural factor, location, education, attitude and knowledge about contraception, cultural beliefs, myths and misconceptions about contraception are some of the factors affecting contraceptive utilization [10,20]. Also, previous studies done on HIV positive women showed that HIV status disclosure, previous contraceptive use, age, drinking habit, and sexual activity are determinant of contraceptive use [6,[21], [22], [23], [24], [25], [26], [27]].

The prevalence of any method contraceptives varies across the countries and within each country. In sub-Saharan Africa, Eswatini and Namibia (both 52%) have the highest level of modern contraceptive prevalence in 2019, whereas South Sudan 4% and Chad 6% had the lowest level [13]. Ethiopia, one of sub-Saharan country, has made remarkable progress in increasing contraceptive prevalence rate over the last 15 years. Despite this success, there is a disparity of contraceptive use rate between regions, urban and rural areas, and younger and older women [17]. Modern contraceptive prevalence rate among married women has increased from 14% in 2005 to 41% in 2019 [28]. Previous studies in the Ethiopia have demonstrated that the rate of contraceptive use ranged between 36.3% in wollega, Ethiopia [29] and 50% in Gondar, Ethiopia [6]. Although there were some studies about contraceptive use in Ethiopia, evidence about contraceptive use among sexually active HIV positive women is limited. Therefore, this study aimed to assess contraceptives prevalence rate and associated factors among HIV positive sexually active women at anti-retroviral therapy clinic at Felege Hiwot Referral Hospital ART clinic in Bahir Dar, Ethiopia.

## 2 Materials and methods

### 2.1 Study area and setting

This study was conducted at Felege Hiwot referral Hospital ART clinic which is located in Bahir Dar City, capital city of Amhara regional State, from June 01^th^ -30^th^, 2018. Bahir Dar City is located 564 km northwest from Addis Ababa, the capital city of the Ethiopia. The Hospital has started providing ART services for free since 2005.the Hospital provides different inpatient and outpatient services to the population of the region including ART and family planning services for clients referred from all district and zonal health care facilities in the Regional State.

### 2.2 Study design and population

An institution-based, cross-sectional study design was used to assess contraceptive utilization of HIV positive women attending ART clinic at Felege Hiwot referral Hospital. The source population for this study was all reproductive age (15–49 years old) living with HIV/AIDS who were attending the ART clinic. A total 11,120 people living with HIV were enrolled in ART of the hospital during the study period. Out of these, 50.76% were reproductive age women. All of them were on HAART at the time of the study. Randomly selected sexually active HIV positive women between 15 and 49 years old, available at the time of data collection, who were sexually active six months prior to the study and who had at least one visit have been included in this study. Sexually active HIV positive reproductive age women with surgical removal of uterus, permanent contraception, who were unable to communicate, who were pregnant, who had a known infertility, received service for less than one month and who were below the age of 18 without family or guardian were excluded from the study.

### 2.3 Sample size and sampling procedure

A total sample of 308 was calculated using single population formula; [n = Z^2^_α/2_× P (1-P)/d^2^] [30]; with the following assumptions. Prevalence of contraceptive utilization P = 76% from previous study conducted in Addis Ababa, Ethiopia [24], (d) margin of error d = 5%, Zα⁄2 = 1.96 at 95% confidence interval and non-response rate of 10%. Study participants were recruited by using simple random sampling method i.e., lottery method. The research team carried out the following activities in the sampling procedure. First, we retrieved the list of female clients from ART service record. Then, the numbers of sexually active HIV positive women aged 15–49 years old were enumerated, and listed based on eligibility criteria, and sampling frame was developed. A code number was given to each eligible participant and was written on a piece of paper. All pieces of papers were placed into a box. Finally, each pieces of paper were picked randomly until the required number of sample size was achieved.

### 2.4 Measurement

#### 2.4.1 Variables

The main outcome variable was self-reported contraceptive use of any method. Independent variables included in this study were age of woman, educational level, and number of living children, drinking habit, and partner HIV status, HIV status disclosure to partner, having stable sexual partner, previous contraceptive use, condom use, and having sexual activity in the last six months.

#### 2.4.2 Operational definitions

**Contraceptive use**: Current use of any method by women to delay or avoid pregnancy for the last six months prior to the study commencement [6].

**Consistent condom use**: The use of female or male condoms in all vaginal sexual relationships with casual and/or steady partners [23].

**Sexual activity**: A woman who had sexual intercourse at list once in the last six months [24].

**Drinking habit**: Drinking of alcohol more than 12 drinks per week [31].

### 2.5 Data collection instrument

The questionnaire had assessed Socio-demographic characteristics, reproductive history, sexual history, contraceptive use and HIV related factors. The questionnaire was developed by organizing variables from previous studies done researches (Additional File 1) [5,23,24]. First, the developed questionnaire was structured, modified and prepared in English language. Then, language experts translated it to local language (*Amharic*) and back to English language. The content validity of the questionnaire was assessed by seven experts from the Bahir Dar University Nursing, Public Health, and Midwifery Departments who had more than four years of research experience each. The internal consistencies of all indexes were assessed, and were found to be satisfactory. The team then pre-tested the questionnaire at Addis Alem Hospital, located in Abay Mado, out of the study area with 30 mothers (10% of the sample size). Based on the pre-test results, the questionnaire wording was modified for clarity. Medical records of participants were also reviewed to get clinical information on anti-retroviral treatment and CD4 cell count.

### 2.6 Data collection and data quality assurance

Trained data collectors have collected the data using the pre-tested interviewer-administered questionnaire at working hours. Four diploma nurses and two Bachelor of Science nurses were recruited as data collectors and supervisors respectively. Additionally, we gave training for data collectors and supervisors on the overall content of questionnaire, ethical issues, and data collection process for two consecutive days. Assigned supervisors have closely managed the data collection process on daily basis.

### 2.7 Data processing and analysis

The collected data were checked manually for completeness and consistency. Then, coded and entered into EPI Info version 3.5.3 and transferred to SPSS version 20 for analysis. Descriptive statistics were used to summarize socio-demographic characteristics of participants and to show prevalence of contraceptive use. Survey logistic regression analysis was carried out at two levels to identify factors associated with contraceptive use. First, bivariate logistic regression was performed to each independent variable with the outcome variable. Then, variables with p-value < 0.05 in bivariate analysis were included in multivariate analysis. Strength of association was measured using odds ratio and 95% confidence intervals. Finally, statistical significance was declared at p-value <0.05.

### 2.8 Ethics approval and consent to participate

Ethical clearance obtained from Bahir Dar University, department of nursing research committee and college of health science institutional review board. Permission letter was obtained from Felege Hiwot Referral Hospital medical director officials. Each study participant was adequately informed about the aim of the study and written consent was sought from each participant or the family/guardian when the participants were less than 18 years of age. Confidentiality was assured by conducting the interview in private rooms which were prepared for this purpose. All the participants’ information was held confidential by locking with keys in the boxes and passwords in computers to avoid access exposing to the third person. Moreover, personal identifiers were not included in the questionnaire.

## 3 Result

### 3.1 Socio-demographic characteristics

All 308 sampled HIV positive women participated in the current study making a response rate of 100%. About one-third of participants 180 (60.1%) of participants were in the age category of 15–34 years, mean age of women was 27 ± 4.8 years, over two third 214 (78.2%) of participants were from Orthodox Christianity. Almost half of participants 164 (53.2%) were educated, 144 (46.8%) participants were uneducated and almost two-third (59.7%) of participants were unemployed. The majority of participants, 260 (84.4%) had drinking habit (Table 1).

**Table 1:** Socio-demographic, Reproductive and sexual characteristics of participants attending ART clinic in FHRH, Bahir Dar City, North west Ethiopia, 2018.

Variable	Category (n = 308)	Frequency	Percent (%)
Age (in years)	15–34	185	60.1
	35–49	123	39.9
Religion	Orthodox	241	78.2
	Muslim	45	14.6
	Others^1^∗	20	6.5
Educational label	Educated	164	53.2
	Uneducated	144	46.8
Marital status	Married	66	21.6
	Unmarried	241	78.4
Occupation	Unemployed	184	59.7
	Government employ	63	20.5
	Private organization employ	61	19.8
Having Monthly income	Yes	181	58.8
	No	127	41.2
Drinking habit	Yes	260	84.4
	No	48	15.6
Number of living children	No child	56	18.2
	One and above children	251	81.8
Child death	Yes	67	21.8
	No	241	78.2
Desire to have child in the future	Yes	140	45.5
	No	168	54.5
Stable sexual relationship	Yes	172	55.8
	No	136	44.2
Had sexual activity in the last 6 months	Yes	172	55.8
	No	136	44.2
Number of sexual partners	None	103	33.4
	One	181	58.8
	Two and above	24	7.8
Changed regular sexual partner	Yes	75	24.4
	No	233	75.6
Reasons for changing	Partner died	19	25.3
	Divorced	26	34.7
	Rejected by spouse	20	40

1∗ = protestant, catholic, Jehovah witness.

### 3.2 Reproductive and sexual characteristics

Nearly one-third 67 (21.8%) of participants had previous child death and less than half of participants (45.5%) had desire to have more children in the future. More than half (55.8%) of participants were sexually active during the last six months before the survey. Similar proportion of women (55.8%) had stable sexual relationship. About one-third, 103 (33.4%) of participants had one partner during the last six months before the survey. About one-fourth of participants (24.4%) had changed their partner since their diagnosis. The main reasons for changing sexual partner were, divorce (34.7%), partner death (25.3%), and 16 (21.3%) spousal rejection (Table 1).

### 3.3 Contraceptive use related features

The prevalence of contraceptive use was 38.3 % (95%CI: 32.5%–43.5%). Almost two-third (64%) of study participants reported that they had previous experience of contraceptive use and only one-fourth (26%) of participants were using dual contraceptive method. The most commonly used contraceptive were injectable (43.5%) followed by pills (21.4) and implant (19.2%). Reasons for choosing particular method were convenience (34.4%), being used secretly (26.9%), no need of more children (20.4%) and used as a dual protection (9.7%)**.** One hundred thirty-six (44.2%) participants were using condom consistently (Table 2).

**Table 2:** Contraceptive use and HIV related features of participants attending ART clinic in FHRH, Bahir Dar City, North west Ethiopia, 2018.

Variable	Category (n = 308)	Frequency	Percent (%)
Ever used contraceptives	Yes	197	64.0
	No	111	36.0
Currently using contraceptives	Yes	118	38.3
	No	190	61.7
Types of preferable methods used	Pills	66	21.4
	Injection	134	43.5
	Implant	59	19.2
	Others^1^∗	49	15.9
Reason for choosing particular method (n = 186)	Convenience	64	34.4
	Cost	16	8.6
	Can be used secretly	50	26.9
	No need more children	38	20.4
	Dual protection	18	9.7
recommend contraception to others	Yes	209	67.9
	No	91	29.5
	Not certain	8	2.6
Use dual contraceptive method	Yes	80	26.0
	No	228	74.0
Currently using condom	Yes	143	46.4
	No	165	53.6
Use of condom in the last 6 months	consistently	136	44.2
	Not consistently	172	55.8
With whom too often use condom?(n = 139)	regular sexual partners	76	54.7
	casual sexual partners	63	45.3
Recent CD4 count (cells/mm3) (n = 142)	<200	12	8.5
	200–349	32	22.5
	350–500	31	21.8
	≥500	67	47.2
Partner tested for HIV	Yes	224	72.7
	No	84	27.3
Partner HIV status (n = 224)	Positive	182	81.3
	Negative	42	18.8
Disclosure of your HIV status to partner	yes	65	21.1
	No	243	78.9
sexual partner change since diagnosis	yes	75	24.4
	No	233	75.6
reasons for changing sexual partner (n = 305)	Partner died	82	26.9
	Divorced	115	37.7
	Spousal rejection	108	35.4
Treated for STI's since HIV diagnosis	Yes	34	11.0
	No	274	89.0

**Others 1∗ =** loop, spermicide, calendar and traditional methods.

### 3.4 HIV related features of participants

Regarding HIV related features of participants, most 67 (47.2%) had a CD4 count ≥500 cells/mm3 and 12 (8.5) had <200 cells/mm3 CD4 count. More than three-fourth (72.7%) participants' partners were tested for HIV and majority (81.3%) were sero-positive. Majority of participants, 243 (78.9%) had not disclosed their HIV status to their partner. The majority of participants (89.0%) had not been treatment for STI's since their HIV diagnosis (Table 2).

### 3.5 Factors associated with contraceptive use

After adjusting confounding factors, maternal age, drinking habit, previous contraceptive use experience, HIV status disclosure to sex partner, and having sexual activity in the last six months remained significant in multivariate logistic regression. Age was significantly associated with contraceptive use. Participants with younger age (15–34) years old were three times more likely to use contraception (AOR = 3.09, 95%CI: 1.59–5.99) compared with older (35–49) age. Likewise, HIV status disclosure to sex partner was significantly associated with contraceptive use. Participants who disclosed their status to their sex partners were almost 2.75-folds more likely to use contraception (AOR = 2.76, 95%CI: 1.14–6.66) compared with their counterparts. Similarly, previous contraceptive use history was significantly associated with contraceptive use. Participants who had previous contraceptive use history were almost 3.36-folds more likely to use contraception (AOR = 3.36, 95%CI: 1.68–6.74) compared with their counterparts. Besides, sexual activity was significantly associated with contraceptive use. Participants who had sexual activity in the last six months prior the study were 5.45-folds more likely to use contraception (AOR = 5.45, 95%CI: 2.72–10.91) compared with participants who had not sexual activity. Nevertheless, drinking habit was negative associated with contraceptive use. Participants who had drinking habit were four times less likely to use contraception (AOR = 4.35, 95%CI: 1.82–10.38) compared with women who did not have drinking habit (Table3).

**Table 3:** Multivariate analysis of factors associated with contraception use among participants attending ART clinic in FHRH, Bahir Dar City, North west Ethiopia, 2018.

Variables	Contraceptive use, n (%)		COR (95%CL)	AOR (95%CL)	p-value
	Yes	No			
Age of woman of woman					
15–34	92 (49.7%)	93 (50.3%)	3.69 (2.19–6.21)	**3.09(1.59- 5.99)∗**	**0.001**
35–49	26 (21.1%)	97 (78.9%)	Ref	Ref	
Educational level of woman					
Educated	73 (44.5%)	91 (55.5%)	1.77 (1.11–2.82)	1.66 (0.92–3.03)	0.095
Uneducated	45 (31.3%)	99 (68.8%)	Ref	Ref	
Drinking habit of woman					
Yes	79 (30.4%)	181 (69.6%)	Ref	Ref	
No	39 (81.3%)	9 (18.8%)	9.93 (4.59–21.47)	**4.35(1.82-10.38)∗**	**0.001**
Previous contraceptive use experience					
Yes	100 (50.8%)	97 (49.2%)	5.33 (2.99–9.48)	**3.36(1.68-6.74)∗**	**0.001**
No	18 (16.2%)	93 (83.8%)	Ref	Ref	
HIV status of tested sexual artner					
Positive	61 (33.5%)	121 (66.5%)	Ref		
Negative	24 (57.1%)	18 (42.9%)	2.65 (1.33–5.24)	1.88 (0.89–4.00)	0.101
HIV status disclosure to partner					
Yes	34 (52.3%)	31 (47.7%)	2.08 (1.19–3.61)	**2.76(1.14,6.66)∗**	**0.024**
No	84 (34.6%)	159 (65.4%)	Ref	Ref	
Having stable sexual partner					
Yes	84 (48.8%)	88 (51.2%)	2.86 (1.75–4.67)	1.85 (0.94–3.64)	0.075
No	34 (25.0%)	102 (75.0%)	Ref	Ref	
Having Sexual activity in the last 6 months					
Yes	91 (52.9%)	81 (47.1%)	4.74 (2.705–7.605)	**5.45(2.724-10.908)∗∗**	**0.000**
No	27 (19.9%)	109 (80.1%)	Ref	Ref	
used condom in the last six months					
Yes	70 (49.0%)	73 (51.0%)	2.34 (1.462–3.738)	1.36 (0.754–2.468)	0.304
No	48 (29.1%)	117 (70.9%)	Ref	Ref	

∗p-value <0.05; ∗∗p-value<0.01. Bolding indicates a significant variable.

## 4 Discussion

Contraceptive use issue among women enrolled in HIV care and treatment programs in the study area has important implications for the health of women and their infants. The most common contraceptive used was injectable (43.5%) followed by pills (21.4) and implant (19.2%). Previous study done in Gondor Hospital, Ethiopia and Nigeria [6,32] have reported similar findings. The current study revealed that only one hundred eighteen (38.3%) sexually active HIV positive reproductive age women were using any method of contraception. The finding was consistent with the study done in wollega, Ethiopia (36.3%) [29] and Kenya (38.5%) [33]. On the other hand, the finding of the current study was lower than evidences from Zambia (69 %) [25], North West Ethiopia (47.7%) [23], Uganda (55.1%) [34], Ireland (55%) [12] Addis Ababa, Ethiopia (43.6%) [22], Tanzania (54%), and Gondar, Ethiopia (50%) [6]. However, the prevalence of this study was higher than the studies done in Southwestern Uganda (27.8%) [21] and western Africa (8%) [12]. Prevalence variation might be due socio-demographic factors, socioeconomic factors, timing of the studies, cultural dissimilarities and differences in health care service utilization of study population and the referenced population.

Many factors have predicted contraceptive use in the current study. Among these factors, HIV positive women with younger age (15–34 years old) were more likely to use contraceptive method compared with older (35–49 years old). Previous studies in Ethiopia, Uganda and Tanzania [6,21,23,35] have reported similar findings. Other evidences have also shown that as the women gets older their need for contraception and rate of contraceptive use decreases [10]. This could be explained in terms of young mothers are usually more open to accept modern technology, often less bound to cultural beliefs that hider contraceptive utilization and better educated than older mothers than older mothers. In addition, younger mothers might regard themselves at risk of unplanned pregnancy while older women might not regard themselves at risk of pregnancy due to their older age. These explanations are supported by studies from Nigeria and Namibia [32,36] which showed that there is a significant association between educational status and contraceptive usage. Older mothers are also affected by cultural and religious beliefs that oppose contraceptive utilization, and they assume that they cannot get pregnant at their perimenopause period [10]. Currently, national and international family planning interventions focus on younger women but the finding of this study suggests that family planning intervention should be directed towards both younger and older women.

Similarly, HIV positive women with previous contraceptive use history were more likely to use contraception compared with their counterparts. The finding is in congruent with studies done in different part of Ethiopia [22,23,37,38] and Uganda [27]. The possible explanation could be due to past contraceptive use experience could minimize myths and misconceptions heard about contraception. There are some myths and misconceptions that hinder women from contraceptive use. For example, “some people believe that implants inserted in the arm could migrate to the brain and causes brain disease including brain cancer and contraceptive use can end up with infertility”. Other misconceptions about family planning include, contraceptive users end up with health problems, contraceptives are dangerous for woman's health and contraceptive use can damage woman's womb [20]. Previous study in urban Africa has demonstrated that negative myths and misconceptions about family planning in the community are barriers to modern contraceptive utilization [20]. This suggests that community based educational programs are needed to dispel common myths and misconceptions about contraception especially for new contraceptive users.

HIV positive women who disclosed their HIV status to their partners had higher odds of using contraception. Previous studies from Ethiopia, Kenya, Nigeria and Zambia [[24], [25], [26],^33^] have reported similar findings. This highlights that disclosure of HIV status to a sexual partner might ease communication between sexual partners to make decision about reproductive issues including contraceptive use. Asfaw et al. [24] has concluded that disclosure of HIV status is important to get support from family especially, husband and discussion can clarify uncertainties about contraceptives and possibly to increase confidence of women. A study from Tanzania [39] has shown that couple-communication facilitates the uptake of contraceptive utilization among HIV-positive reproductive age women. Another study in Tigray region, Ethiopia [5] has also indicated that open discussion with husbands/sexual partners about contraceptive methods was positively associated with contraceptive utilization. Moreover, a study in Uganda [40] has revealed that women who did not disclose their HIV status to their sexual partners were less likely to use contraception. This implies that health care providers in ART clinics must discuss with clients about disclosure of HIV status to their sexual partners.

Having Sexual activity in the last six months prior to the commencement of the study also was significantly associated with contraceptive use. Participants who had sexual activity in the last six months prior to the study were 5-folds more likely to use contraception compared with participants who had not sexual activity. The finding is in agreement with studies from Ethiopia and Zambia and Sub-Saharan Africa [22,25,41]. The possible explanation could be HIV positive women who are engaged in sexual activity have concern about the risk of pregnancy if they don't use contraceptive methods. A previous study in Sub-Saharan Africa [41] has indicated that the odds of contraceptive use was increased for women who had unprotected sex either with partner or outside partner. This suggests that women who are engaged in sexual activity should be appropriately counseled for family planning and provided with accessible contraceptive methods.

On the other hand, HIV- positive reproductive age women who had drinking habit were less likely to use contraceptive methods. The result is in line with the study conducted in Uganda [34]. This highlights that alcohol can affect women's judgment and memory. A woman consuming excessive amount of alcohol may forget to take the pill or use condom at that day. Previous studies in Britain and United States of America [31,42] have demonstrated that women having drinking habit are usually engaged in risky sexual behaviors that expose to unwanted pregnancy and sexually transmitted infection. A systematic review by Terplan et al. [43] has also indicated that women with substance use disorders including alcohol used contraception less often than non-substance-users. Another study from USA [44] revealed that risk drinking is related to ineffective contraception and condom use. The finding suggests that women with drinking habits should be provided with appropriate long acting contraceptive methods or be counseled to avoid the daily basis contraceptive methods.

### 4.1 Limitation

This study has some limitations. First, the study was cross-sectional in nature so that cause and effect relationship cannot be established. Second, men were not included in the study in spite of they have important role to play in deciding about fertility issues and family size. Third, as fertility issue is a sensitive topic, social desirability bias cannot be avoided.

## 5 Conclusion

A significant proportion of HIV positive women had low level of contraceptive use that was lower than the national recommended level. Injectable was the most preferred method of contraception while convenience was the main reason for contraceptive use. Participants with younger age (15–34years), who disclosed HIV status to sex partner, had history of contraceptive use, and who were sexually active six months prior to the study were more likely to use contraception. However, participants who had drinking habit were less likely to use contraception. Integration of family planning service with ART service and increased attention to women having drinking habit are recommended. These results suggest that multi-sectorial and multi-disciplinary approaches are needed to increase contraceptive use in the HIV positive women. Lastly, interventional and longitudinal studies are needed to improve contraceptive use among HIV positive reproductive age women.

## Declarations

### Author contribution statement

T. Tewabe: Conceived and designed the experiments; Performed the experiments; Wrote the paper.

T. Ayalew: Performed the experiments; Analyzed and interpreted the data; Wrote the paper.

D. Jenbere, M. Ayehu, G. Talema and A. Abdanur: Conceived and designed the experiments; Performed the experiments.

E. Asmare: Conceived and designed the experiments; Contributed reagents, materials, analysis tools or data.

### Funding statement

This research did not receive any specific grant from funding agencies in the public, commercial, or not-for-profit sectors.

### Declaration of interests statement

The authors declare no conflict of interest.

### Additional information

No additional information is available for this paper.
