# Supplementary material for: Contraceptive use and associated factors among sexually active reproductive age HIV positive women attending ART clinic at Felege Hiwot Referral Hospital, Northwest Ethiopia: A cross-sectional study
Source: Heliyon. 2020 Dec 14;6(12):e05653. doi: 10.1016/j.heliyon.2020.e05653 (PMC7736717; doi:10.1016/j.heliyon.2020.e05653)
Supplement: Supplementary file 1 — Additional File 1 [file mmc1.docx]

**Annex I: QUESTIONNAIRE**

**Part One: Demographic variable of participant**

| NO | Questions | response | code | skip |
| --- | --- | --- | --- | --- |
| 101 | How old are you? | --------- years(age in complete years) |  |  |
| 102 | Religion | 1. Orthodox  2. Catholic  3. Protestant  4. Moslem  5. Others |  |  |
| 103 | Educational level | 1. Illiterate(can’t read and write)  2. Able to read and write (no grade)  3. Primary  4. Secondary  5. Tertiary or university |  |  |
| 104 | Marital status | 1. Single 2.Married  3. widowed 4. Cohabiting partner  5. Divorced 6.Separated |  |  |
| 105 | Main Occupation | 1. Unemployed  2. Government employ  3. Private organization employ  4. Day laborer  5. Commercial sex workers  6. Student  7. Other ------------ |  |  |
| 106 | Do you have monthly  Income? | 1.yes 2.no |  |  |
| 107 | Total monthly income |  |  |  |
| 108 | Drink alcohol | 1.yes 2.no |  |  |

**Part two : Contraceptive and condom use among the study participants.**

| NO | Questions | response | code | skip |
| --- | --- | --- | --- | --- |
| 209 | Have you ever use  Contraception? | 1.yes 2.no |  |  |
| 210 | Are you currently using contraception method? | 1.yes 2.no |  |  |
| 211 | Do you use more than one method of contraception? | 1.yes 2.no |  |  |
| 212 | What main contraceptive  Methods are you using? | 1. Pills 2. Injection 3. Implant 4. Condom 5. Others |  |  |
| 213 | Why was that particular method chosen? | 1. Convenience 2. Cost (it is cheap) 3. Can be used secretly 4. Other |  |  |
| 214 | Would you recommend contraception to others? | 1. Yes 2. no 3. Not certain |  |  |
| 215 | Have you ever used condoms? | 1.yes  2.no |  |  |
| 216 | How would you best describe your use of condoms in the last 6 months? | 1.Always use (consistently use)  2.Often use  3.Sometimes use  4.Rarely use |  |  |
| 217 | When do you often use condoms? | 1. With regular sexual partners  2. With casual sexual partners |  |  |
| 218 | Have you ever heard of dual protection? | 1.yes 2.no |  |  |
| 219 | Do you think HIV positive couples should consistently use condoms? | 1.yes 2.no |  |  |
| 220 | Why should HIV positive persons use condoms? | 1. Prevent re-infection  2. Prevent STDs  3. Prevent infecting their sexual partners |  |  |

**Part three: Clinical and social factor of participants**

| NO | Questions | response | code | Skip |
| --- | --- | --- | --- | --- |
| 321 | Are you currently on ARVs? | 1.yes 2.no |  |  |
| 322 | Duration on ARVs | 1.Write number  2. I can’t remember  3.I don’t know |  |  |
| 323 | Your CD4 number is | 1.Write number  2. I can’t remember  3.I don’t know |  |  |
| 324 | Do you know the HIV status of your regular Partner /partners? | 1.yes 2.no |  |  |
| 325 | If yes what was the result? | 1.Positive  2.Negative |  |  |
| 326 | Did you disclose your status to your sexual partner? | 1.yes 2.no |  |  |
| 327 | Have you changed regular sexual partners since HIV diagnosis? | 1.yes 2.no |  |  |
| 328 | If they have changed partners, what were the reasons? | 1. Partner died 2. Divorced   3. Rejected by spouse  4.Other reasons |  |  |
| 329 | Are you treated for STD since HIV diagnosis? | 1.yes 2.no |  |  |

**Part four: Reproductive and sexual factor of participant**

| NO | Questions | response | code | Skip |
| --- | --- | --- | --- | --- |
| 430 | How many children do you have? | _________ |  |  |
| 431 | Have any of your children died? | 1.yes 2.no |  |  |
| 432 | Do you intend to have any more children? | 1.yes 2.no |  |  |
| 433 | Do you have stable sexual relationship? | 1.yes 2.no |  |  |
| 434 | Did you had sex in the last 6 months? | 1.yes 2.no |  |  |
| 435 | How many sexual partners have you had in the last 6months? | 1. None 2. One   3.Two  4.Three  5.More than 3 |  |  |
| 436 | Does your regular partner know your HIV status? | 1.Yes  2.No  3.Don’t know |  |  |
